# Electrocatalytic Coupling Conversion of Methane by Dual‐Site Control in Nickel Oxyhydroxide

**DOI:** 10.1002/advs.76137

**Published:** 2026-06-15

**Authors:** Kailong Lu, Yangshen Chen, Junhao Wang, Yi Zhang, Sunyang He, Huimin Xu, Hui Liu, Jiao Deng

**Affiliations:** ^1^ Suzhou Institute of Nano‐Tech and Nano‐Bionics Chinese Academy of Sciences Suzhou China; ^2^ School of Nano‐Tech and Nano‐Bionics University of Science and Technology of China Hefei China; ^3^ Nano Science and Technology Institute University of Science and Technology of China Suzhou China

**Keywords:** dual active site, electrocatalysis, electronic overlap, methane conversion, nickel oxyhydroxide

## Abstract

Methane (CH_4_), as both a carbon resource and greenhouse gas, requires an effective and ecologically friendly catalysis to accomplish the conversion into valued‐added chemicals or fuels, wherein the establishment of a proper multi‐site active structure to boost the C─H dissociation and C─C coupling demands further exploration. Herein, we disclose a facile and general approach by controlling the electronic property of Ni to preserve a moderate orbital hybridization (d‐p) with O, and thereby induce a synergistic Ni─O dual‐site for the expected electrocatalysis of CH_4_ toward C_2_ product. Specifically, along the d band shifting downwards via the Ni valence increase, the optimal NiOOH not only affords sufficient capability for the CH_4_ oxygenation relative to the Ni(OH)_2_, but also minimizes the probability of O detachment compared with the NiO_2_. The resultant NiOOH catalyst exhibits the highest Faradaic efficiency of 53.5% with a production rate of 401.6 µmol g_cat._^−1^ h^−1^ or 1.61 µmol h^‒1^ cm^‒2^ for acetic acid, and a long‐term stability for over 50 h without detectable structural deterioration. This study develops an efficient electrocatalytic CH_4_ process, and could arouse extensive attention to the new materials and routes meeting the economicity and sustainability.

## Graphical abstract

Electrocatalytic coupling conversion of methane (CH_4_) is accomplished on the nickel oxyhydroxide (NiOOH), wherein the Ni─O dual‐site is triggered via the proper electronic interaction, synergistically promoting the C─H activation and C─C formation, enabling a selective and efficient C_2_ product generation route under ambient conditions.

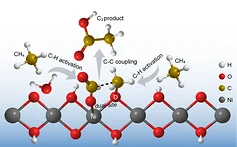

## 1 Introduction

Methane (CH_4_), the essential component of natural gas, serves as an important carbon feedstock and energy to the petroleum industry, and also behaves as a greenhouse gas with the global warming potential (GWP) exceeding 80‐times that of carbon dioxide (CO_2_) over a 20‐year horizon [1, 2, 3]. To pursue the economic benefit and actualize the carbon‐neutrality target, converting CH_4_ to chemicals or fuels in an efficient and mild way has become a concerned topic in both scientific research and application development [4, 5]. The physicochemical inertness feature of the CH_4_ molecule, such as low polarizability and high C─H bond energy (439 kJ mol^‒1^), induces CH_4_ activation via a two‐step indirect route (steam reforming and Fischer‐Tropsch synthesis) or a one‐step direct route (partial oxidation), which typically operate with severe conditions (e.g., temperatures > 600°C) [6, 7, 8, 9]. Electrocatalysis, a representative and emerging pathway for CH_4_ conversion in recent years, has been discovered to exploit the electric potential to overcome the kinetic barrier of C─H polarization and dissociation under ambient conditions [10, 11]. Beyond the effective CH_4_ activation, a smooth passage for the C─C coupling to generate the value‐added C_2_ products would meet the superior applied demand, where the pivotal factor is the design and tuning of catalytic structures to form the synergistic dual‐sites, and thereby manage the relevant carbon‐involved intermediate behavior [12, 13]. Existing approaches generally construct the differential active sites through the multi‐composition structures located in the single architecture (doping/alloying) [14, 15, 16] or two constitution (heterojunction) [17, 18], accompanied by the unfavorable character of material complexity and mechanistic understanding. Consequently, seeking an alternative solution to create the dual‐site synergy in one basic structure alone, and simultaneously investigating the effect of their interaction on the CH_4_ catalytic process, deserves an innovative attempt.

Besides the electronic interchange between the dual‐sites across the aforementioned multi‐compositions, inspired by the Hubbard Band Theory (HBT), metal (oxy)hydroxides attract attention that the inner d‐p orbital hybridization could stimulate the metal‐oxygen interaction (M─O) as the catalytic dual‐sites within the single materials [19, 20, 21, 22]. One of the representatives, nickel oxyhydroxide (NiOOH), has been developed in the electrochemical oxidation of ammonia, methanol, and other slightly larger organic molecules (glycerol or urea), etc. [23, 24, 25, 26, 27, 28]. Not only does the intrinsic oxidizing power of NiOOH drives the efficient conversion of these small molecules, but also the structural and electronic regulation, accompanied by the reaction condition optimization, would affect and alter their reaction pathways, and eventually work through the issues regarding product selectivity, overoxidation, and side reaction. Taking a feasible solution into the CH_4_ oxidation, especially, the initially conceived M─O interaction, NiOOH exposes abundant surface metal and oxygen sites, and preserves the high‐valence Ni (+3) to supply the sufficiently deep d‐band to overlap with the p‐band of O, which triggers the Ni─O dual‐site synergetic mode for catalysis [29, 30]. The strong‐correlation electronic behavior in NiOOH has been considered to be more common relative to that inside the nickel hydroxide (Ni(OH)_2_), where the low‐valence Ni (+2) affords deficient d‐p band mixing with the O, and thereby it is hard to activate the O atom as another catalytic site in addition to the Ni site [31]. Notably, excessive d‐p coupling when the valence of Ni further increases would raise the risk of O escape from the matrix to enhance the occurrence probability of the side reaction and also bring the stability issue [32]. Along this working principle, the intention is to regulate the d‐p (Ni─O) interaction at a proper stage, while taking advantage of the derived dual‐site to facilitate the CH_4_ activation followed by the coupling step within the catalytic reaction, which remains to be validated through a comprehensive and detailed research.

In this work, the NiOOH material was prepared by an electrochemical synthesis method for the CH_4_ electrocatalysis. Through adjusting the electrochemical procedures, the resultant samples can be controlled as Ni(OH)_2_, NiOOH, or their composites. Structural and electronic characterization verified the distinguishable features of formal valence (+3/+2) and atomic bonding (Ni─O) between the NiOOH and Ni(OH)_2_ with a similar morphology and crystalline. These varying properties prompted the enhancement of CH_4_ adsorption and activation on the NiOOH relative to the Ni(OH)_2_. The optimum Faradaic efficiency (FE) for C_2_ product (acetic acid) of NiOOH was 53.5%, which is over 9 times that of Ni(OH)_2_. Simultaneously, the NiOOH catalyst can steadily operate for at least 50 h with a high FE retention rate of 92.5%. In situ characterizations and theoretical simulations disclose the catalytic nature that the intensive d‐p band overlap along the oxidation state increase of the Ni atom initiated the lattice O atom, and the Ni─O dual‐site cooperatively reduced the energy barrier for the activation and coupling of the paired CH_4_ molecules. Furthermore, the sample with a strong O vacancy formation tendency by the overmuch d band shift (NiO_2_), exhibited a low electrocatalytic activity toward CH_4_ conversion. This study demonstrates an effective valence/band tuning approach for the construction of the multi‐active sites to realize the favorable C─C coupling process, and it may act as a universal guidance for the future search of the CH_4_ catalysis system.

## 2 Results and Discussion

### 2.1 Material Preparation and Characterization

**FIGURE 1:**
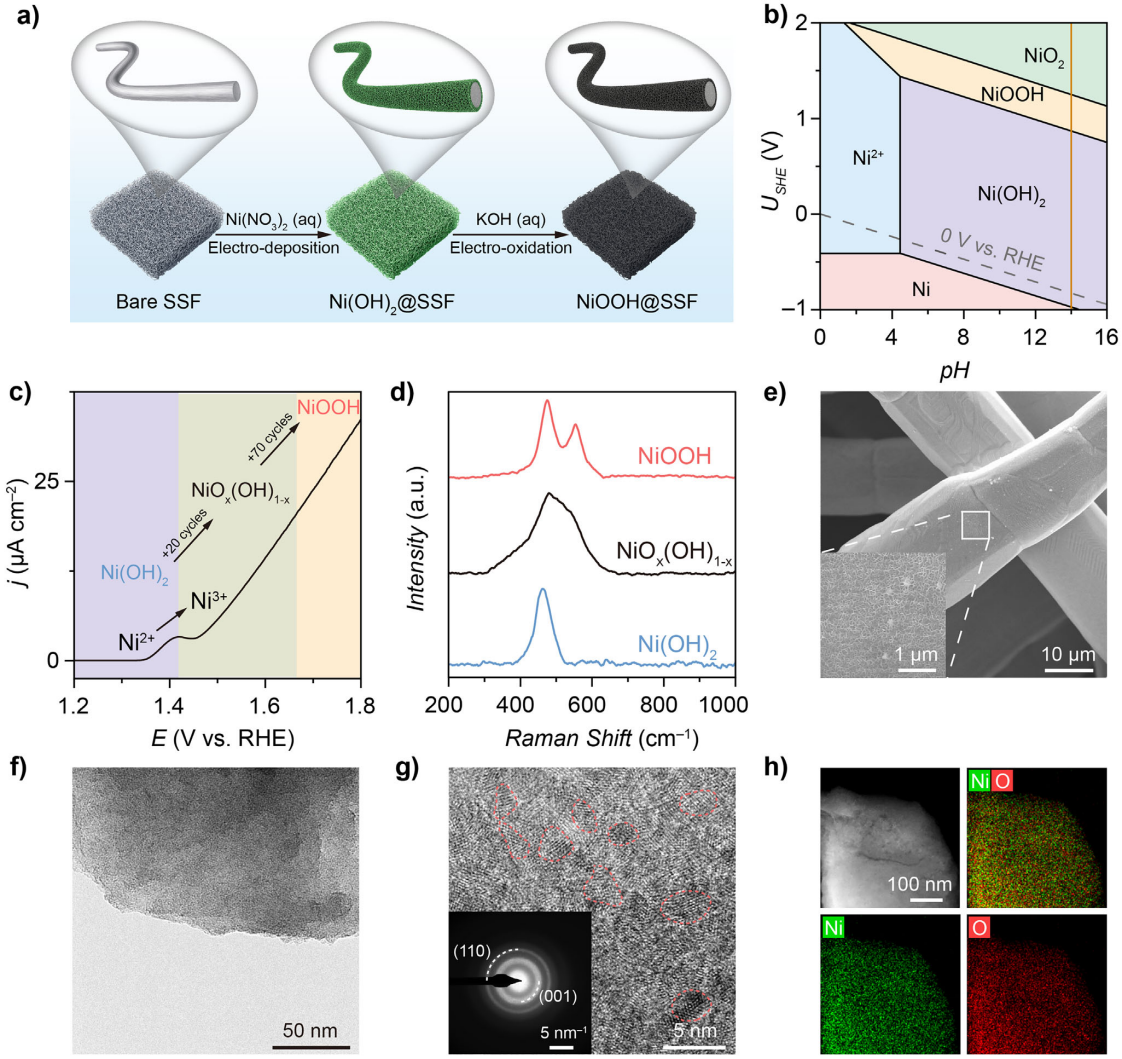
(a) Schematic illustration of the NiOOH synthesis on the SSF substrate, including the electro‐deposition to obtain the Ni(OH)_2_ followed by the electro‐oxidation treatment. (b) Pourbaix diagram of various Ni‐based materials, displaying their thermodynamic stability regions as a function of pH and applied potential. (c) LSV scans in 1.0 m KOH to convert the Ni(OH)_2_ to NiOOH, and the Ni^2+^/Ni^3+^ redox peak was observed. (d) Raman spectra of Ni(OH)_2_, NiOOH, and the sample at the incomplete transformation stage. (e) SEM image of NiOOH with a porous and sponge‐like morphology on the substrate, the inset shows a magnified SEM image revealing the interconnected pores and stacked nanosheets. (f) TEM image of NiOOH with a nanosheet structure. (g) HRTEM image of NiOOH, the inset shows the crystalline nanodomains with lattice fringes by red dashed outline, and the SAED patterns with the diffuse diffraction rings assigned to the characteristic (001) and (110) planes by white dashed line. (h) High‐angle annular dark‐field scanning transmission electron microscopy (HAADF‐STEM) image and the corresponding EDS elemental mapping of Ni and O atoms of NiOOH.

The electro‐deposition with Ni(NO_3_)_2_ aqueous solution (pH ≈ 4.5) was conducted at room temperature to synthesize the Ni(OH)_2_ material on the stainless‐steel fiber (SSF) substrate, and then the electro‐oxidation treatment in KOH (1 m) electrolyte was carried out to obtain the NiOOH material (Figure 1a and Methods section in Supporting Information). On the basis of the Pourbaix diagram [33] presenting the thermodynamically stable regions of Ni(OH)_2_ and NiOOH (Figure 1b), the linear sweep voltammetry (LSV) scans across the potential range of 1.2–1.8 V versus RHE were implemented repeatedly to induce the Ni(OH)_2_‐to‐NiOOH transformation (Figure 1c). The predominant oxidation peak at ∼1.45 V versus RHE, attributed to the Ni^2+^/Ni^3+^ redox pairs, suggests the occurrence of the internal composition variation beyond this potential threshold [34]. Raman spectroscopy was utilized to monitor the transition progress (Figure 1d). The Ni(OH)_2_ exhibited a characteristic single band at 461 cm^‒1^ (Ni^2+^─O), and it was replaced by the doublet peaks at 478 cm^‒1^ and 558 cm^‒1^ after 90 LSV cycles, which come from the Ni^3+^─O vibration of NiOOH [35, 36]. The mixed stage with the overlapping signals of Ni^2+^─O and Ni^3+^─O (e.g., 20 cycles) highlights the importance of the electrochemical protocol in controlling the structural integrity of the NiOOH. Scanning electron microscopy (SEM) image revealed a porous and sponge‐like morphology of the NiOOH uniformly coating on the SSF arrays (Figure 1e; Figure S1), similar to the architectural character of the Ni(OH)_2_ before LSV scans (Figure S2). The abundant interconnected channels are expected to expose sufficient surface‐accessible Ni─O sites and facilitate the mass transport for the electrocatalysis. Transmission electron microscopy (TEM) images displayed the analogous nanosheets distributed within the NiOOH and Ni(OH)_2_ (Figure 1f; Figures S3 and S4a). High‐resolution TEM (HRTEM) images showed that these nanosheets were composed of several randomly oriented nanocrystalline (2–5 nm) with definite lattice fringes, which derived the symmetric diffraction patterns in the fast Fourier transform (FFT) images (Figure 1g; Figures S4b and S5a–e). Selected area electron diffraction (SAED) patterns exhibited the diffuse diffraction rings of the (001) and (110) planes, further verifying the short‐range order nature of the NiOOH (inset of Figure 1g; Figure S5f). Yet, X‐ray diffraction (XRD) patterns revealed no observable crystal peaks in both NiOOH and Ni(OH)_2_ (Figure S6), implying that the relatively small nanocrystalline with an irregular orientation would lead to the long‐range disorder feature within the NiOOH nanosheets [37, 38, 39]. Notably, the structural order decreases in the NiOOH compared to that in the Ni(OH)_2_, likely stemming from the reduced symmetry within the Ni─O octahedron by the Jahn‐Teller distortion during the Ni^2+^‐to‐Ni^3+^ oxidation [40]. Energy‐dispersive X‐ray spectroscopy (EDS) elemental mapping image indicated the homogeneous distribution of the Ni and O atoms inside the NiOOH framework (Figure 1h). The maximal preservation of the pristine structure when the NiOOH transforms from the Ni(OH)_2_ according to the above results, warrants a central study on the Ni─O band regulation for the electrocatalytic CH_4_ conversion.

**FIGURE 2:**
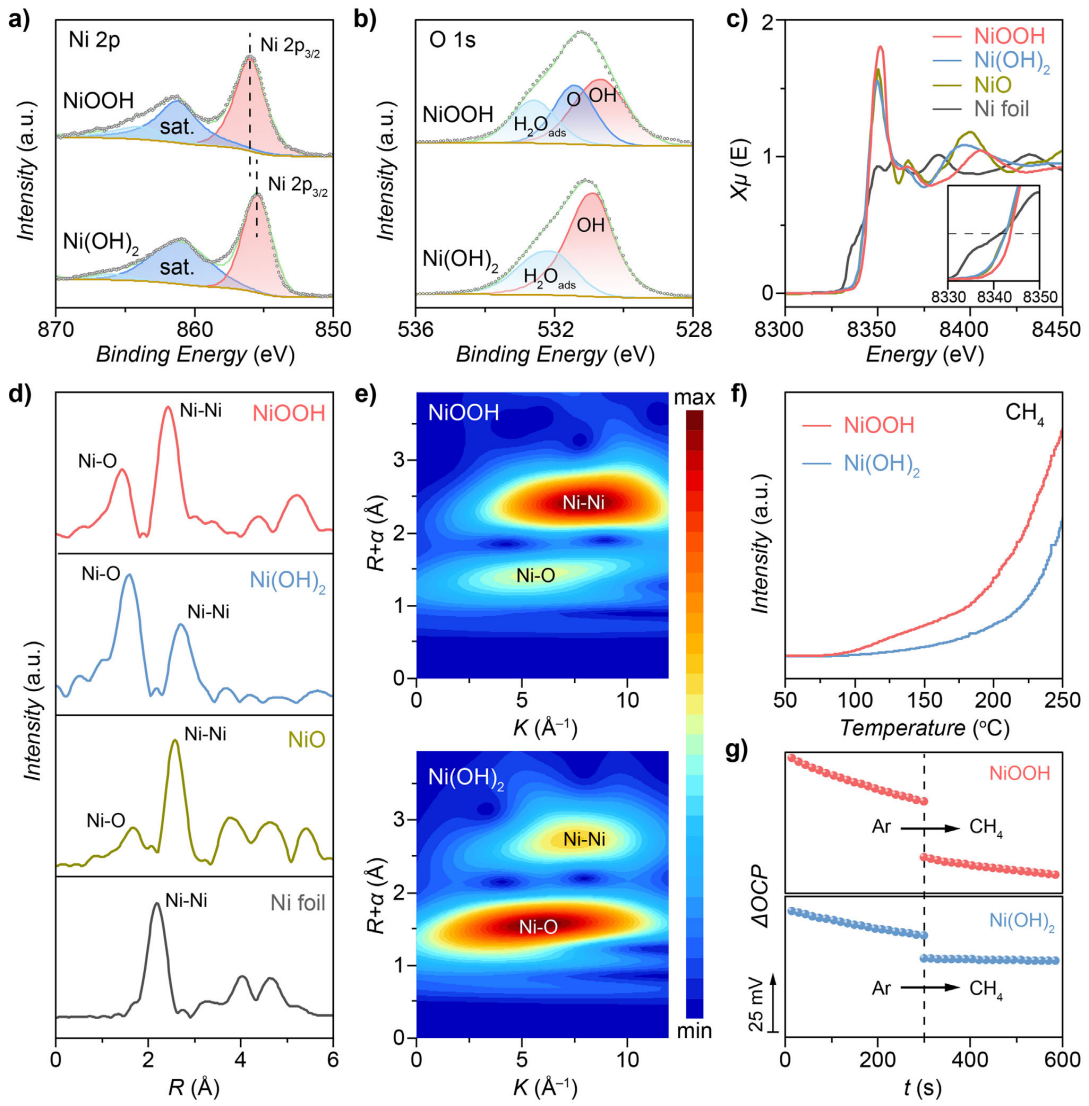
(a) Ni 2p_3/2_ XPS spectra of NiOOH and Ni(OH)_2_. (b) O 1s XPS spectra of NiOOH and Ni(OH)_2_. (c) Normalized Ni K‐edge XANES spectra of NiOOH in comparison to Ni(OH)_2_, NiO, and Ni foil, the inset shows the absorption edge by zooming in the energy region between 8330 and 8350 eV. (d) Fourier transform magnitude of the k^3^‐weighted Ni K‐edge EXAFS spectra of NiOOH in comparison to Ni(OH)_2_, NiO, and Ni foil. (e) Ni K‐edge WT‐EXAFS spectra of NiOOH and Ni(OH)_2_. (f) CH_4_‐TPD plots of NiOOH relative to Ni(OH)_2_. (g) Open circuit potential variation (ΔOCP) tests via switching the imported gas from Ar to CH_4_ on NiOOH relative to Ni(OH)_2_.

Spectroscopic techniques, including X‐ray photoelectron spectroscopy (XPS) and synchrotron radiation‐based X‐ray absorption spectroscopy (XAS), were utilized to examine the electronic and structural properties of the NiOOH and Ni(OH)_2_. The Ni 2p XPS spectra displayed a primary peak centered at 856.0 eV attributed to the characteristic Ni^3+^ 2p_3/2_ spectroscopic term of NiOOH, while the Ni^2+^ 2p_3/2_ peak of Ni(OH)_2_ at 855.4 eV, accompanied by the corresponding satellite peaks (Figure 2a) [41]. The binding energy shift toward the high‐valence Ni hints the d‐band variation and structural transition between the NiOOH and Ni(OH)_2_. Through deconvolution of the O 1s XPS spectra, a Ni─O signal at 531.4 eV exists in addition to the Ni─OH (530.7 eV) and absorbed H_2_O (H_2_O_ads_, 532.5 eV) signals in the NiOOH (Figure 2b) [42]. Compared to the Ni(OH)_2_, the extra Ni─O bond and the decrease of the Ni─OH intensity within the NiOOH, guarantee the hydroxide‐to‐oxyhydroxide transformation to afford the unoccupied O (deprotonation) nearby the Ni as the surface Ni─O dual‐site for the subsequent catalysis. Ni K‐edge X‐ray absorption near‐edge structure (XANES) spectra further approved the oxidation state increase of Ni by the positive‐shift of the absorption edge and the elevation of the white line peak in the NiOOH relative to the Ni(OH)_2_ (Figure 2c). Extended X‐ray absorption fine structure (EXAFS) of Ni K‐edge via Fourier‐transformation (Figure S7) displayed two typical coordinate shells (Ni─O and Ni─Ni) of the NiOOH and Ni(OH)_2_ (Figure 2d). The shortened Ni─O (1.59 to 1.44 Å) and Ni─Ni (2.70 to 2.42 Å) distances likely originated from the valence increase and thereby the enhanced electrostatic interaction among the Ni─O sites of NiOOH, benefit the d‐p coupling behavior across the material matrix. This shift tendency of the Ni─O and Ni─Ni bonds inside the NiOOH and Ni(OH)_2_ can be visualized by the wavelet transform (WT) EXAFS (Figure 2e), and the inverse intensity relationship (Ni─O versus Ni─Ni), as a result of the varied local structural disorder, intuitively differentiates their respective bonding state [43, 44].

Chemical and electrochemical adsorption/desorption analyses were performed to detect the CH_4_ adsorption capability of the NiOOH and Ni(OH)_2_ with the different electronic structures. The CH_4_ temperature programmed desorption (TPD) tests were conducted before the breakdown temperature (<250°C) of the materials (Figure 2f). Along the temperature rise, there appeared a visible CH_4_ desorption profile and an increasing desorbed amount at ∼150°C on the NiOOH in contrast to the Ni(OH)_2_, implying the stronger intrinsic CH_4_ affinity of the NiOOH. The open circuit potential variation (ΔOCP) responding to the adsorbate‐induced charge redistribution in the electric double layer [45], was extracted through switching the imported gas from Ar to CH_4_ in the electrolyte (Figure 2g). At the mutation point during the continuous operation (300 s), the ΔOCP of the NiOOH was much more significant than that of the Ni(OH)_2_, suggesting more CH_4_ adsorption on the material surface under the electrochemical working environment. These results rationalize the efficacy of the Ni─O dual‐site with a reinforced electronic interaction inside the NiOOH to the CH_4_ adsorption, supplying a prerequisite for the CH_4_ activation of the following electrocatalysis.

### 2.2 Electrocatalytic Evaluation

**FIGURE 3:**
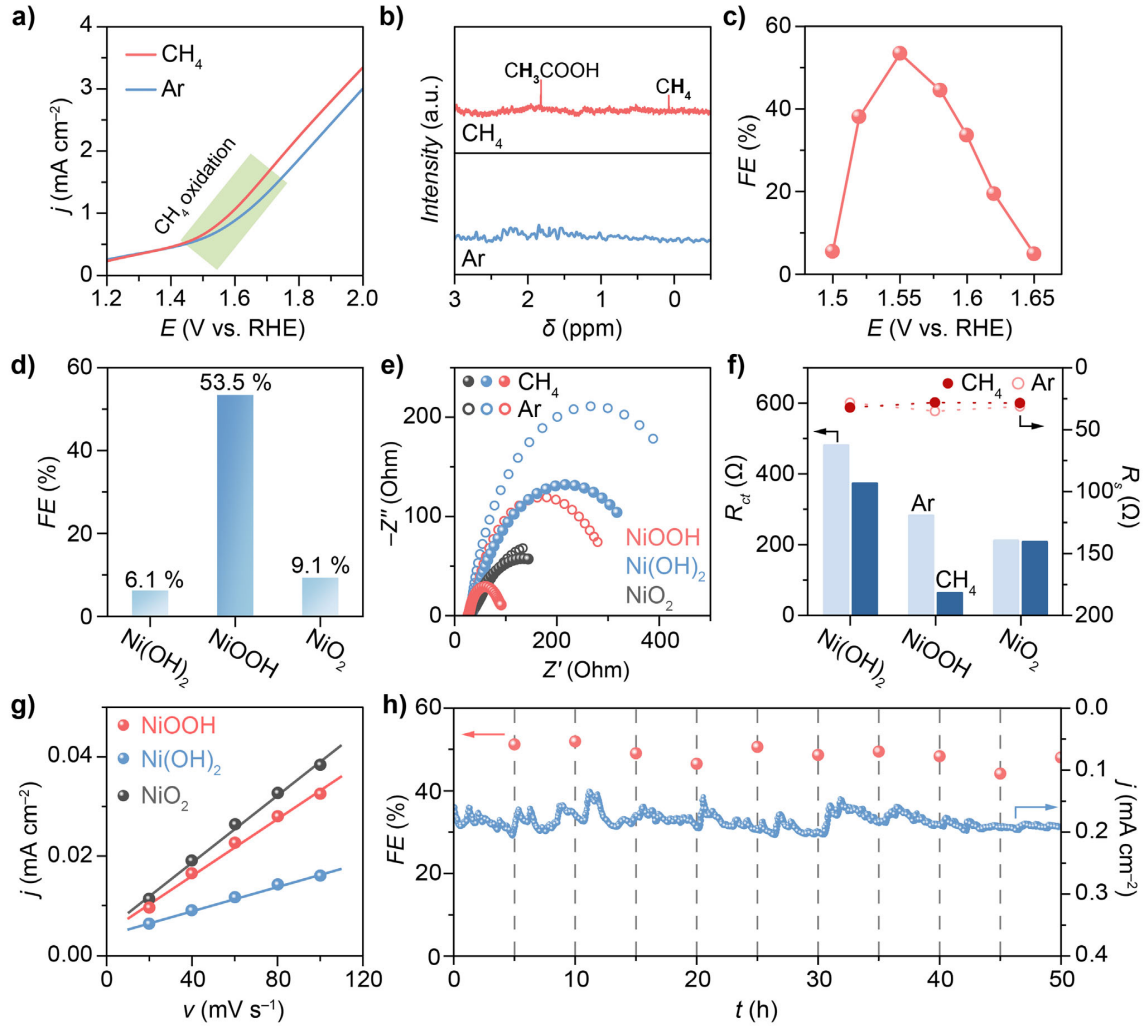
(a) LSV curves of NiOOH in 0.1 m K_2_CO_3_ electrolyte saturated with CH_4_ and Ar gas. (b) ^1^H NMR spectra of the electrolyte after electrochemical tests with NiOOH under CH_4_ and Ar atmosphere at 1.55 V versus RHE. (c) FE of acetic acid production over NiOOH as a function of applied potential across 1.5 to 1.65 V versus RHE. (d) Optimal FE of acetic acid production for Ni(OH)_2_, NiOOH, and NiO_2_ at 1.55 V versus RHE. (e) EIS Nyquist plots of Ni(OH)_2_, NiOOH, and NiO_2_ in Ar and CH_4_ saturated 0.1 m K_2_CO_3_. f) Statistical comparison of charge transfer resistance (R_ct_, bars) and ohmic resistance (R_s_, lines) derived from the EIS fitting in figure e. (g) ECSA measurements of Ni(OH)_2_, NiOOH, and NiO_2_. (h) Long‐term stability test of NiOOH for 50 h at 1.55 V versus RHE.

Electrochemical measurements were carried out in a flow cell with 0.1 m K_2_CO_3_ electrolyte (Figure S8 and Methods section in Supporting Information), and all the applied potentials were converted into the reversible hydrogen electrode (RHE). Linear sweep voltammetry (LSV) curves in CH_4_‐saturated and Ar‐saturated electrolytes of the NiOOH revealed a distinct difference in polarization current density beyond 1.5 V versus RHE (Figure 3a), implying the catalytic conversion of CH_4_ on the catalyst surface. The slight current increment between the CH_4_ and Ar atmosphere on the Ni(OH)_2_ hints its inferior activity (Figure S9). Bulk electrolysis via chronoamperometry was implemented with CH_4_ gas under ambient pressure, and the solution electrolytes were quantitatively analyzed through the ^1^H nuclear magnetic resonance (^1^H NMR) spectroscopy by employing the external reference method (Figure S10). The only acetic acid (CH_3_COOH) product was identified at the chemical shift of 1.81 ppm in addition to the residual CH_4_ at 0.05 ppm within the detection limit, while there exists none of any signals with Ar gas at the same reaction conditions (Figure 3b; Figure S11). Across the electrochemical test scope of 1.5 to 1.65 V versus RHE, the highest FE of 53.5% with a production rate of 401.6 µmol g_cat._^−1^ h^−1^ or 1.61 µmol h^‒1^ cm^‒2^ for the C_2_ product was obtained on the NiOOH at 1.55 V versus RHE (Figure 3c). In contrast, the optimum detected FE of the Ni(OH)_2_ (6.1%) was much lower (Figure 3d), consistent with the recorded LSV trend (Figure 3a; Figure S9). The significant activity promotion of CH_4_ conversion to C_2_ product (CH_3_COOH) by the NiOOH versus Ni(OH)_2_ and their composites at the incomplete transformation stage (Figure S12), verifies the positive effect of the strengthened d‐p band overlap via the valence electron change (Ni^3+^ and Ni^2+^) on the Ni─O dual‐site synergy for the C─H activation and C─C coupling processes. Yet, for the NiO_2_ possessing a similar layered structure (Figures S13 and S14), the FE considerably decreases to 9.1%, presumably as a result of the over‐activated lattice oxygen to induce the side reaction (e.g., oxygen evolution) when the further d‐p interaction happens (Ni^4+^─O), indicating the necessity of an appropriate d‐p hybridization control inside the catalyst (here the NiOOH) to balance the reactivity and stableness of the Ni─O structure toward the highly‐efficient electrocatalysis of CH_4_.

Electrochemical impedance spectroscopy (EIS) was employed to assist in understanding the surface active site behavior among the various catalysts (Figure 3e). Through fitting the Nyquist plots by the equivalent electrical circuit (EEC) model (Figure S15 and Table S1), the ohmic resistance (R_s_) of the electrochemical loop maintained at an equal level for all the materials with either CH_4_ or Ar gas (Figure 3f), guaranteeing a primary study on the catalytic interface. The charge transfer resistance (R_ct_), the feedback of the electrocatalytic kinetics, presented a successive reduction tendency (Ni(OH)_2_ > NiOOH > NiO_2_) under Ar atmosphere (Figure 3f), which could be an indirect proof of the valence rising (Ni^2+^ → Ni^3+^ → Ni^4+^) to enhance the d‐p electronic coupling, and subsequently supply the growing Ni─O concerted sites for the interfacial charge exchange. This active site‐related order was in accord with the electrochemical active surface area (ECSA) analysis (Figure 3g; Figure S16). When the gas constituent was replaced by CH_4_ (Figure 3f), the R_ct_ of the NiOOH dropped significantly, while a much smaller decrease appeared in the Ni(OH)_2_, supporting the priority of the activated Ni─O surface within the NiOOH along the reaction channel of CH_4_ activation. In addition, there exists a comparable R_ct_ inside the NiO_2_, clarifying the unfavourableness of the hyperactive Ni─O lattice to the electrocatalytic CH_4_ reaction. Electrochemical durability evaluation by the potentiostatic mode (1.55 V versus RHE) was continuously conducted under CH_4_ atmosphere, and the electrolyte was sampled every 5 h for the quantitative determination (Figure 3h). The FE of CH_3_COOH product and reaction current density on the NiOOH can be maintained around their initial state for over 50 h (10 cycles), e.g., the detected FE value of 49.5%, which can be mathematically converted as the retention rate of 92.5%. After the long‐term operation, no detectable structural deterioration of the catalyst occurred through the Raman spectra and XPS spectra characterizations (Figures S17 and S18), validating the system reliability of the electrocatalytic CH_4_ conversion by the NiOOH catalyst. The attractive activity and stability lead to a benchmark of the numerous electrocatalysts so far (Table S2), and our material lies among the first‐rate ones in terms of the FE value for the C_2_ product with its steady period, while there is a significant room for the improvement of the production rate to keep up with some of these statistical catalysts in the future.

### 2.3 In Situ Analysis

**FIGURE 4:**
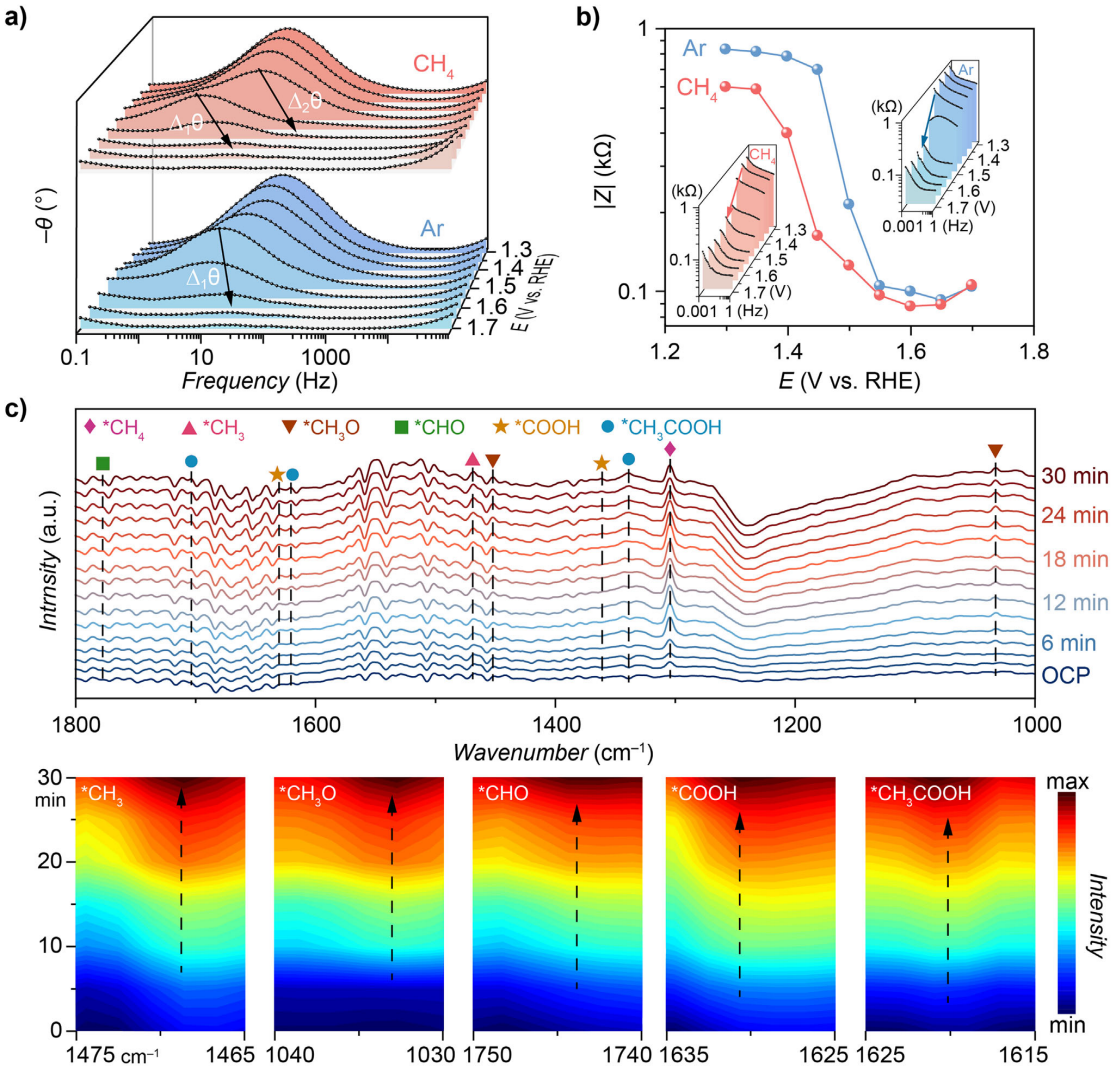
(a) Potential‐dependent Bode plots (phase angle versus frequency) of NiOOH in Ar and CH_4_ saturated 0.1 m K_2_CO_3_. (b) Potential‐dependent |Z| at 0.001 Hz for NiOOH in Ar and CH_4_ saturated 0.1 m K_2_CO_3_, the insets show the corresponding recorded Bode plots (|Z| versus frequency) for the data extraction. (c) Time‐dependent in situ ATR‐FTIR spectra on the NiOOH surface in 0.1 m K_2_CO_3_ at 1.55 V across the wavenumber of 1000 to 1800 cm^‒1^. The intermediates of *CH_3_, *CH_3_O, *CHO, *COOH, and *CH_3_COOH along the time lapse are shown individually by the 2D contour plots.

In situ characterization techniques, containing the in situ EIS and attenuated total reflectance Fourier transform infrared (ATR‐FTIR) spectroscopy, were adopted to monitor the reaction trace and intermediate evolution on the NiOOH surface at working conditions. The potential‐dependent EIS spectra were recorded within the potential of 1.3 to 1.7 V versus RHE under CH_4_ and Ar atmospheres (Figure 4a,b). The Bode plots by phase angle (θ) versus potential across the mid‐frequency range (1–1000 Hz) showed a single θ drop tendency (Δ_1_θ) starting at 1.55 V versus RHE in an Ar environment, while an extra θ falling (Δ_2_θ) happened at 1.5 V versus RHE in a CH_4_ atmosphere (Figure 4a). The two separate Δ_1_θ and Δ_2_θ suggest the existence of different reaction channels, i.e., oxygen evolution reaction (OER) and methane oxygenation reaction (MOR) [46, 47]. The shift toward a lower potential region of the Δ_2_θ compared to the Δ_1_θ, indicates the faster startup of MOR relative to OER driven by the NiOOH material, and thereby leads to the successive activity increase between 1.5 and 1.55 V versus RHE (Figure 3c). Beyond 1.55 V versus RHE, the more persistent signal of Δ_1_θ under CH_4_ atmosphere implies the continuously aggravated OER as the dominate option considering the inferior mass transport of CH_4_, which supports the activity decrease trend from 1.55 to 1.65 V versus RHE (Figure 3c). In the Bode plots by impedance modulus (|Z|) versus potential at the low‐frequency range (0.001–1 Hz) which can feedback the diffusion behavior of substance (Figure 4b) [48, 49], the |Z| drop (Δ|Z|) along the potential raise appeared both in CH_4_ and Ar environment, as a result of the accelerated reactant consumption when the driving force enhanced nearby the catalytic interface. The negative shift (horizontal direction) of the Δ|Z| profile in the CH_4_ case (red) in comparison to the Ar case (blue), also hints the prior occurrence of the MOR within the NiOOH catalyst.

The in situ ATR‐FTIR detection to capture the fingerprints related to the procedures of CH_4_ dissociation, oxygenation, and coupling, was conducted under the electrolysis at 1.55 V versus RHE by importing CH_4_ gas into 0.1 m K_2_CO_3_ for 30 min with NiOOH (Figure 4c). A series of constantly increasing signals came out after the pre‐electrolysis at open‐circuit potential (OCP) over the measured wavenumber of 1000–1800 cm^‒1^, which can be clearly visualized by the contour maps along the vertical time axis (Figure 4c; Figure S19) [50, 51, 52]. The *CH_4_ peak at 1305 cm^‒1^ verifies the necessary adsorbed reactants for the electrocatalysis. Next, the *CH_3_ (1468 cm^‒1^), *CH_3_O (1033 and 1452 cm^‒1^), and *CHO (1778 cm^‒1^) signals corroborate the CH_4_ dehydrogenation and oxygenation steps after the C─H activation. Then, the C═O stretching vibration at 1363 and 1631 cm^‒1^ designates the *COOH species as the terminated oxidation group of one CH_4_ molecule, and afterwards coupled with another *CH_3_ species from the second dissociated CH_4_ molecule to ultimately yield the *CH_3_COOH (1340, 1621, and 1704 cm^‒1^). On the basis of the above intermediate tracking for the CH_4_‐to‐CH_3_COOH conversion, a comprehensive catalytic cycle with the stepwise transition of the structure and reactant could be proposed on the synergetic Ni─O dual‐site inside the NiOOH catalyst (Figure S20). These in situ analyses not only deepen the understanding of the experimental product trajectory but also serve as guidance to the theoretical reaction coordinate establishment.

### 2.4 DFT Calculations

**FIGURE 5:**
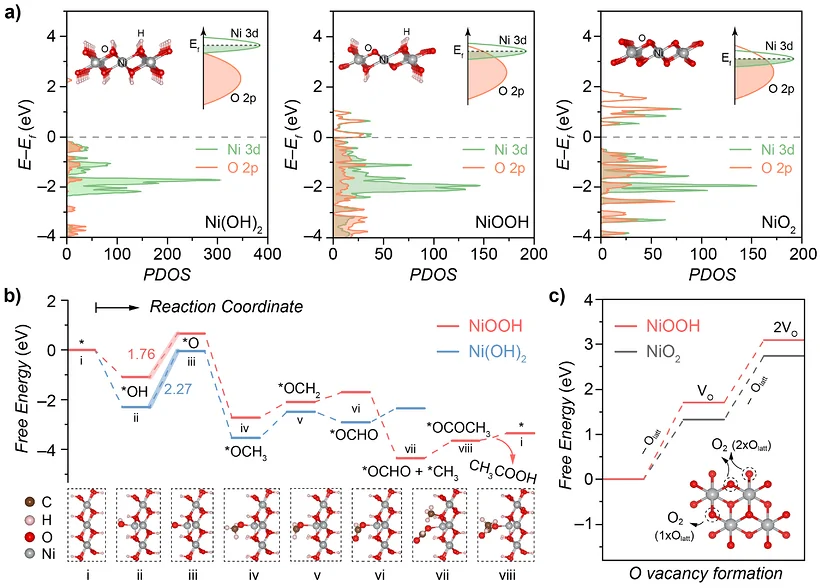
(a) DOS calculation of Ni 3d and O 2p bands for Ni(OH)_2_, NiOOH, and NiO_2_, the insets show the corresponding calculated models and schematic illustration of d‐p hybridization. (b) Free energy for each step along the reaction coordinate of NiOOH and Ni(OH)_2_, the insets show the corresponding atomic structures with the adsorbed intermediates for the calculations. (c) Free energy profiles of V_o_ formation over NiOOH and NiO_2_, the inset shows the schematic illustration of one and two lattice oxygen (O_latt_) detachment to participate in the side reaction of oxygen evolution.

Theoretical simulation by density functional theory (DFT) calculations was implemented to supply a mechanistic insight into the effect of electronic property tuning within the Ni─O dual‐site on the catalytic process. In view of the catalyst structure as the long‐range disordered nanosheet composed of the short‐range ordered nanocrystalline by the experimental characterizations, the typical periodic slab models by exposing the (001) planes were exploited for the computation (inset of Figure 5a; Figure S21), which also refers to a class of research regarding the amorphous or weak crystalline systems [53, 54, 55]. This detected (001) plane (inset of Figure 1g) was generally adopted by the various reported studies to simulate the layered transition metal oxyhydroxide materials [23, 56, 57], where the in‐plane Ni─O coordinate network is parallel to the two‐dimensional (2D) matrix (a‐axis and b‐axis), and the interlayer stacking is along the c‐axis to be perpendicular to the 2D surface (Figure S21). In addition, the (001) plane was examined to be the most stable one among a series of low‐index crystal planes after sufficient structural relaxation and optimization (Table S3). To clarify, the theoretical models established here were mainly utilized to focus on the local Ni─O dual‐site related electronic property and energy calculations, while not to reflect the entire architecture experimentally. Through the density of states (DOS) computation, a visible downshift of the d‐band center following the gradual increase of Ni oxidation state in Ni(OH)_2_ (‒1.440 eV), NiOOH (‒1.976 eV) and NiO_2_ (‒2.233 eV) was observed (Figure 5a). There exists a progressive growth of the unoccupied O p‐band above the Fermi level, and a continuous enlargement of the overlapped region between the occupied Ni d‐band and O p‐band, which indicate the Ni─O d‐p hybridization was enhanced from Ni(OH)_2_ to NiOOH, and to NiO_2_, rationalizing the original intention based on the Hubbard Band Theory (HBT) [19]. Assisted by the ATR‐FTIR for the intermediate assignment, the free energy (ΔG) for each step along the reaction coordinate were calculated, accompanied with the corresponding atomic structures (i‐viii) to display the adsorption status of various species on the NiOOH model (Figure 5b). By single‐point energy calculation comparison amongst the essential structures and reaction steps, there appeared a limited influence from the possible surface polarity, a dipole moment perpendicular to the 2D matrix, to the obtained results (Table S4), and thereby the data without correction were presented. In terms of the thermodynamic ΔG, the reaction steps of CH_4_ activation, dehydrogenation, oxygenation, and coupling (iii → viii) become quite smooth (all ΔG < 0.7 eV) after the formation of surface *O at the Ni site (ii → iii) with the maximal barrier inside the NiOOH. Taking account of the electrochemical driving force (>1.5 V versus RHE) experimentally, the much lower barrier of 1.76 eV within the NiOOH relative to 2.27 eV of the Ni(OH)_2_ (ii → iii) reflects the better reactivity of the Ni site. Meanwhile, another CH_4_ dissociation step at the lattice O site (vi → vii) of NiOOH proceeds favorably in comparison to the Ni(OH)_2_ case as well. These pivotal steps associated with the C─H activation and C─C coupling at the Ni and O sites, presenting as a superior state for the NiOOH versus Ni(OH)_2_, demonstrate that the reinforced d‐p electronic interaction could synergistically promote the Ni─O dual‐site for the electrocatalytic MOR. Through benchmarking the energy barriers of the active site (*O) formation step (ii → iii) and CH_4_ activation step (iii → iv; vi → vii), the minimal values (‒3.39 and ‒2.66 eV) for the two steps of *CH_4_ to *CH_3_, in addition to a feasible step of *OH to *O on the NiOOH were realized (Table S5), supporting the competitive and favorable nature of NiOOH in contrast to the reported catalysts. Further raise of the d‐p hybridization (NiOOH to NiO_2_) may initiate the excessive activation of the lattice O to be detached while leaving the oxygen vacancy (V_o_) amongst the matrix (Figure S22) [58, 59]. The computed formation energies of one and two V_o_ for NiO_2_ are 1.33 and 2.74 eV, while 1.7 and 3.1 eV for NiOOH accordingly (Figure 5c), illustrating the stronger tendency toward OER side reaction with the participation of lattice O inside the NiO_2_. Overall, the interpretations of DFT calculations are consistent with the experimental phenomenon, validating the initial principle that the moderate d‐p interaction by adjusting the electronic structures will optimize the Ni─O dual‐site synergy, and eventually maximize the activity and stability of CH_4_ electrocatalysis into C_2_ product on the NiOOH catalyst. Notably, this balance between the active site improvement and the structural deterioration by the excessive regulation has also been endorsed in the electrocatalytic oxidation of other small molecules [60, 61, 62].

## 3 Conclusions

In summary, the laminal NiOOH nanosheet material has been developed by the electrochemical synthesis for the electrocatalytic CH_4_ to C_2_ product. The highest FE for the acetic acid attains 53.5% at 1.55 V versus RHE, and it can maintain a well durability for at least 50 h (10 cycles) with a FE retention rate of 92.5%. Comprehensive consideration of the FE and steady operation time toward the C_2_ product generation, this electrocatalyst belongs to the top‐ranking materials, while its production rate (401.6 µmol g_cat._^−1^ h^−1^ or 1.61 µmol h^‒1^ cm^‒2^) demands significant upgradation. The established structure‐activity relationship by the experimental and theoretical studies demonstrates that tuning the electronic hybridization between the Ni d band and O p band to a proper level will facilitate the Ni─O dual‐site to synergistically activate two CH_4_ molecules on one hand, and confront the lattice O detachment induced side reaction and structural instability on the other hand. Integrally, these surface sites for the optimization of CH_4_ adsorption, transformation, and coupling steps result in the detected performance promotion by the NiOOH catalyst. The gained knowledge in this study, together with future tracking of the multi‐site behavior under the operando conditions, would broaden the search for more, but not limited to, oxyhydroxide materials and novel sustainable CH_4_ conversion pathways.

## Conflicts of Interest

The authors declare no conflicts of interest.

## Supporting information




**Supporting File**: advs76137‐sup‐0001‐SuppMat.docx.

## Data Availability

The data that support the findings of this study are available from the corresponding author upon reasonable request.
