# Supplementary material for: Electrocatalytic Coupling Conversion of Methane by Dual‐Site Control in Nickel Oxyhydroxide
Source: Adv Sci (Weinh). 2026 Jun 15;13(51):e76137. doi: 10.1002/advs.76137 (PMC13336415; doi:10.1002/advs.76137)
Supplement: Supplementary file 1 [file ADVS-13-e76137-s001.docx]

**Supporting Information**

**Electrocatalytic coupling conversion of methane by dual-site control in nickel oxyhydroxide**

Lu, *et al.*

**Methods**

**Chemicals**

Nickel(II) nitrate hexahydrate (Ni(NO_3_)_2_·6H_2_O, AR), potassium hydroxide (KOH, AR), potassium carbonate (K_2_CO_3_, AR), nickel(II) chloride hexahydrate (NiCl_2_·6H_2_O, AR), acetone (C_3_H_6_O, ≥99.8%), ethanol (C_2_H_6_O, ≥99.7%), and isopropanol (C_3_H_8_O, ≥99.7%) were purchased from Sinopharm Chemical Reagent Co., Ltd. 1H,1H,2H,2H-perfluorooctyltrimethoxysilane (C_11_H_13_F_13_O_3_Si, 97%) was purchased from Shanghai Aladdin Biochemical Technology Co., Ltd. Deuterium oxide (D_2_O, 99.9 atom% D) was purchased from Innochem Co., Ltd. Maleic acid (C_4_H_4_O_4_, ≥99%) was purchased from Sigma-Aldrich. Stainless steel felt (SSF) was custom-made by Maipengchen Electronics, Kunshan, China. Argon (99.999%) and methane (99.999%) were supplied by Suzhou Tianping Jinyuan Gas Co., Ltd. All deionized (DI) water was obtained from a Millipore Milli-Q water purification system. Unless otherwise specified, all chemicals were used as received without further purification.

**Materials synthesis**

**Preparation of hydrophobic substrate.** Stainless steel fiber felt (2 x 2 cm) was ultrasonicated sequentially in acetone, ethanol, and DI water for 30 min each time, and then dried at 80 °C. Hydrophobic treatment was conducted through spraying the substrate with 100 µL of C_11_H_13_F_13_O_3_Si solution (1% v/v in isopropanol) and subsequently drying at 80 °C.

**Synthesis of Ni(OH)_2_ and NiOOH.** Ni(OH)_2_ was cathodically electrodeposited onto the pretreated stainless steel fiber substrate via galvanostatic mode at 20 mA for 45 min in a three-electrode electrochemical system with 0.08 M Ni(NO_3_)_2_. The as-prepared material was rinsed with DI water and dried at 80 °C. NiOOH was synthesized by electrochemical oxidation of the Ni(OH)_2_ in 1.0 M KOH electrolyte through LSV cycling (90 cycles, 1.2-1.8 V vs. RHE, scan rate: 20 mV s^−1^). The final material was obtained after rinsing with DI water and drying at 80 °C.

**Synthesis of NiO_2_.** NiO_2_ was prepared via a precipitation method according to the literature.^[1]^ Briefly, 1.0 M NaOH was added to 0.5 M NiCl_2_ solution (pH = 12) at 80℃. The precipitate was treated by centrifugation, washing with DI water, drying at 80 °C, and calcining at 150 °C for 12 h to obtain the final material. For electrode fabrication, the ink with NiO_2_ powder (8 mg), ethanol (500 µL), and Nafion solution (10 µL, 5 wt%) was drop-cast onto the pretreated stainless steel fiber substrate and dried at 80 °C.

**Materials characterization**

Raman spectra were recorded on an inVia Qontor microscope with 532 nm laser excitation. Scanning electron microscope (SEM) was conducted on a Regulus8230 field-emission scanning electron microscope. Transmission electron microscopy (TEM), high-resolution TEM (HRTEM), selected area electron diffraction (SAED), and energy-dispersive X-ray spectroscopy (EDS) mapping were performed on a Tecnai G2 F20 instrument operated at 200 kV. X-ray diffraction (XRD) patterns were collected on a D8 Discover diffractometer using Cu Kα radiation (λ = 1.5406 Å). X-ray photoelectron spectroscopy (XPS) measurements were carried out on a Thermo ESCALAB 250Xi spectrometer. Methane temperature-programmed desorption (CH_4_-TPD) tests were conducted on a TriStar II 3020 surface area and porosity analyzer. X-ray absorption spectroscopy (XAS) experiments were performed through the transmission mode using a Si(111) double-crystal monochromator.

**Electrochemical measurement**

All electrochemical experiments were conducted at room temperature (25 ± 2 °C) in a custom flow cell with a three-electrode configuration using a CHI 760E electrochemical workstation. The as-prepared samples served as the working electrode, platinum wire as the counter electrode, and Hg/HgO electrode as the reference electrode. The anodic and cathodic compartments were separated by a Nafion 117 membrane. Both compartments were filled with a 0.1 M K_2_CO_3_ aqueous solution as the electrolyte. Before each measurement, the reactant gas (CH_4_ or Ar) was purged through the cell at 15 mL min^‒1^ for 15 minutes to saturate the electrolyte. The potentials were converted to the reversible hydrogen electrode (RHE) scale by the equation:

E_RHE_ = E_Hg/HgO_ + 0.098 V + 0.0591 × pH

Electrochemical impedance spectroscopy (EIS) tests were performed over a frequency range from 10^5^ to 10^‒1^ Hz. Electrochemical active surface area (ECSA) was analyzed by the cyclic voltammetry sweeps at scan rates of 20, 40, 60, 80, and 100 mV s ^‒1^.

**Products analysis**

The liquid products in the anodic chamber were quantified using ^1^H NMR spectroscopy (Bruker NMR600). For each ^1^H NMR analysis, 300 µL of the post-electrolysis solution was mixed uniformly with 100 µL of 1 mM maleic acid solution (internal standard) and 100 µL of D_2_O.

The Faradaic efficiency (FE) of the liquid products was calculated according to:

$$\text{FE = }\frac{\text{n × F ×}\text{ }\text{C}_{\text{product}}\text{ ×}\text{ V}_{\text{solution}}}{\text{I}_{\text{average}}\text{ × t}}\text{ × 100\%}$$

Where *n* is the number of electrons transferred per molecule of product, *F* is the Faraday constant (96485 C mol^‒1^), *C_product_* is the product concentration (mol L^‒1^), *V_solution_* is the volume of the electrolyte (L), *I_average_* is the average current during electrolysis (A), and *t* is the reaction time (s).

The production rate based on the catalyst mass loading was calculated as:

$$\text{Production rate = }\frac{\text{C}_{\text{product }}\text{× }\text{V}_{\text{solution}}}{\text{m}_{\text{cat. }}\text{× t}}$$

where *C_product_* is the product concentration (mol L^‒1^), *V_solution_* is the volume of the electrolyte (L), *m_cat._* is the catalyst mass loading (g), and *t* is the reaction time (h).

The production rate based on the electrode area was calculated as:

$$\text{Production rate = }\frac{\text{C}_{\text{product }}\text{× }\text{V}_{\text{solution}}}{\text{t}\text{ }\text{× A}}$$

where *C_product_* is the product concentration (mol L^‒1^), *V_solution_* is the volume of the electrolyte (L), *t* is the reaction time (h), and *A* is the electrode area (cm^‒2^).

**Electrochemical ATR-SEIRAS measurement**

In situ attenuated total reflection surface-enhanced infrared absorption spectroscopy (ATR-SEIRAS, Bruker INVENIO S) was performed using a custom-built three-electrode electrochemical cell. A silicon prism coated with a 100 nm Au film served as the working electrode, a Hg/HgO electrode as the reference electrode, and a carbon rod as the counter electrode. The electrolyte was a 0.1 M K_2_CO_3_ aqueous solution. The catalyst ink was drop-cast onto the Au film. Measurements were conducted in an optical chamber integrated with the spectrometer. Spectra are presented as absorbance (*A*), defined as *A* = −log(*I*/*I*_0_), where *I* and *I_0_* represent the irradiation intensity of incident and reflective beams.

**DFT calculations**

All calculations were conducted using the Cambridge Sequential Total Energy Package (CASTEP) in a command-line interface.^[2]^ The generalized gradient approximation (GGA) with the Perdew-Burke-Ernzerhof (PBE) functional was employed to describe the exchange-correlation interactions.^[3]^ Ultrasoft on-the-fly generated (OTFG) pseudopotentials were used, and a plane-wave cutoff energy of 450 eV was applied.^[4, 5]^ The Broyden-Fletcher-Goldfarb-Shanno (BFGS) scheme was selected as the minimization algorithm.^[6, 7]^ Van der Waals interactions were corrected using the Grimme DFT-D2 correction scheme.^[8, 9]^ A vacuum spacing of 15 Å was applied along the surface normal (Z direction) to avoid the spurious interactions between the periodic images. The convergence tolerance of energy was 2×10^‒5^ eV per atom, the maximum force was 0.05 eV/Å, the maximum stress was 0.l GPa, and the maximum displacement was 0.002 Å.^[10, 11]^ All the models have undergone sufficient relaxation and optimization without the residual traces of obvious crystal collapse, distortion, and reconstruction. Dipole correction was applied in the .param file by setting DIPOLE_CORRECTION to SELFCONSISTENT, and DIPOLE_DIR to Z (c-axis).

The surface energy (γ) was calculated according to the following equation:

$$\text{γ}\text{ }\text{=}\text{ }\frac{\text{E}\text{slab}\text{ }\text{-}\text{ }\text{n ×}\text{ E}_{\text{bulk}}}{\text{2 × A}}$$

Where *E_slab_* is the total energy of the relaxed slab model, *E_bulk_* is the energy of the bulk, *n* is the number of atoms contained in the surface system, and *A* is the area of the surface.

The Gibbs free energy change (ΔG) is defined as:

ΔG = ΔE + ΔZPE – TΔS

where *ΔE* is the electronic energy difference obtained from DFT calculations, *ΔZPE* and *ΔS* are the zero-point energy difference and the entropy change between the products and reactants, respectively, and *T* is the temperature (298.15 K).

The oxygen vacancy formation energy (E_f_) was calculated by the equation:

E_f_ = E_defect_ + n_O_E_O_ – E_perfect_

where *E_defect_* and *E_perfect_* are the total energies of the defective and perfect structures, respectively, *n_O_* is the number of removed O atoms, and *E_O_* is the chemical potential of O, taken as half of the total energy of an O_2_ molecule. A positive E_f_ represents an instability, while a negative value depicts the stability against its constituents.

**Figure S1.** SEM images of NiOOH at different scales, showing a porous and sponge-like morphology on the substrate, which consists of interconnected nanosheets.

**Figure S2.** SEM images of Ni(OH)_2_ at different scales, showing a porous and sponge-like morphology on the substrate, which consists of interconnected nanosheets.

**Figure S3.** TEM images at different scales of NiOOH with a nanosheet structure.

**Figure S4.** (a) TEM image of Ni(OH)_2_ with a nanosheet structure. (b) HRTEM image of Ni(OH)_2_ showing the crystalline nanodomains with lattice fringes, and the inset shows the corresponding FFT patterns of the selected regions by white dashed rectangle.

**Figure S5.** (a-e) HRTEM images of NiOOH, the inset shows the crystalline nanodomains with lattice fringes by red dashed outline, and the corresponding FFT patterns of the selected regions by white dashed rectangle. (f) SAED patterns of NiOOH, the inset shows the diffuse diffraction rings assigned to the characteristic (001) and (110) planes by white dashed line.

**Figure S6.** XRD patterns of NiOOH, Ni(OH)_2_, and bare substrate, showing only the characteristic diffraction signals of the austenitic stainless steel matrix of SSF within all three samples.

**Figure S7.** Ni K-edge EXAFS *k*-space curves of NiOOH (a) and Ni(OH)_2_ (b) with the corresponding numerical fitting.

**Figure S8.** Schematic diagram (a) and physical photograph (b) of the flow-type cell for the electrochemical measurements, including the gas in/out ports, working electrode (WE, NiOOH), reference electrode (RE, Hg/HgO), proton exchange membrane (Nafion-N117), and counter electrode (CE, Pt).

**Figure S9.** LSV curves of Ni(OH)_2_ in 0.1 M K_2_CO_3_ electrolyte saturated with CH_4_ and Ar gas, respectively.

**Figure S10.** a) ^1^H NMR spectra of acetic acid solutions with gradient concentrations (0.05-0.50 mmol), including the maleic acid as the internal standard. b) Calibration curve of acetic acid concentration (*c*) versus the ^1^H NMR peak integral area ratio (*ratio*) of acetic acid to maleic acid. The fitted linear equation serves as the quantitative basis for determining the FE of acetic acid in the electrocatalysis.

**Figure S11.** ¹H NMR spectra of the electrolyte after electrochemical test with NiOOH under CH_4_ atmosphere at 1.55 V vs. RHE, including the maleic acid as the internal standard for the quantification.

**Figure S12.** Optimal FE of acetic acid production at 1.55 V vs. RHE for Ni(OH)_2_ (0 cycle), NiOOH (90 cycle), and other materials with the different LSV scan cycles over the Ni(OH)_2_.

**Figure S13.** XRD pattern of NiO_2_, showing the characteristic diffraction peaks of (010), (011), (012), (110), (220), (200), and (201) planes, consistent with the standard JCPDS card (96-900-9113) and reported results.^[1]^

**Figure S14.** TEM (a) and HRTEM (b) images of NiO_2_, showing a layered structure.

**Figure S15.** EIS Nyquist plots of Ni(OH)_2_ (a, b), NiOOH (c, d), and NiO_2_ (e, f) with the corresponding fitting curves by the equivalent electrical circuit (EEC) model in CH_4_ and Ar saturated 0.1 M K_2_CO_3_.

**Figure S16.** CV curves at different scan rates of Ni(OH)_2_ (a), NiOOH (b), and NiO_2_ (c) in 0.1 M K_2_CO_3_ electrolyte for the ECSA determination.

**Figure S17.** Raman spectra of the NiOOH catalyst before and after 50 h electrolysis.

**Figure S18.** Ni 2p XPS spectra of the NiOOH catalyst before and after 50 h electrolysis.

**Figure S19.** 2D contour plots of the time-dependent in situ ATR-FTIR spectra on the NiOOH surface in 0.1 M K_2_CO_3_ at 1.55 V, showing the species of *CH_4_, *CH_3_, *CH_3_O, *CHO, *COOH, and *CH_3_COOH along the timelapse.

**Figure S20.** Schematic illustration of the methane (CH_4_) to acetic acid (CH_3_COOH) transformation on the NiOOH surface. Grey sphere: Ni atom, red sphere: O atom, brown sphere: C atom, and white sphere: H atom.

**Figure S21.** Calculated structural models with unit cell axes of Ni(OH)_2_, NiOOH, and NiO_2_ by exposing (001) plane. Upper panel: top view, lower panel: side view, and the lattice constants of the constructed supercells are set as a = b = 11.1951 Å.

**Figure S22.** Calculated structural models of NiOOH (a, b) and NiO_2_ (c, d) with the different oxygen vacancy (V_o_) configurations, the dashed circles mark the oxygen vacancy locations inside the matrix.

**Table S1.** EIS fitting parameters (R_s_, R_ct_) of Ni(OH)_2_, NiOOH, and NiO_2_ under Ar or CH_4_ atmosphere.

| Sample | Atmosphere | R_s_ (Ω) | R_ct_ (Ω) |
| --- | --- | --- | --- |
| Ni(OH)_2_ | Ar | 28.32 | 480.3 |
|  | CH_4_ | 32.14 | 372.5 |
| NiOOH | Ar | 35.07 | 281.7 |
|  | CH_4_ | 28.04 | 63.43 |
| NiO_2_ | Ar | 31.27 | 211.3 |
|  | CH_4_ | 28.50 | 207.7 |

**Table S2.** Comparison of FE, production rate for C_2_ products, and steady operation time between this work and reported studies.

| **Catalyst** | **FE_C2_**  **[%]** | **Production rate**  **[μmol g_cat._^−1^ h^−1^]** | **Production rate**  **[μmol h^−1^ cm^−2^]** | **Stability**  **[h]** | **Ref.** |
| --- | --- | --- | --- | --- | --- |
| **NiOOH** | **53.5** | **401.6** | **1.61** | **50** | **This work** |
| Ni/NiO | 89.0 | 25 | 0.87 | 24 | ^[12]^ |
| Mg-MOF-74 | 6.9 | 126.6 | \ | 2.8 | ^[13]^ |
| NiO@NiHF | 85.0 | \ | \ | 3 | ^[14]^ |
| Cu_2_O/CuO | 21.1 | 126.7 | 0.32 | 8 | ^[15]^ |
| WO_3_ | 50.7 | 125090 | 100.07 | 12 | ^[16]^ |
| ZnO nanosheets | 10.0 | 347310 | 277.80 | 0.5 | ^[17]^ |
| V_3_O_7_·H_2_O nanorods | 9.7 | 41.1 | \ | 8 | ^[18]^ |
| CuO_x_/V_2_O_5_ | 21.0 | \ | \ | 6 | ^[19]^ |
| Fe-N-C SAC | 68.0 | 11480.6 | 1.28 | 3 | ^[20]^ |
| Cu/Al_2_O_3_ | 90.0 | \ | 33.50 | 25 | ^[21]^ |
| Mo-Cu-CTAC | 54.5 | \ | 529.10 | 20 | ^[22]^ |
| FeNi(OH)_x_ | 87.0 | 9090 | 0.93 | 2.5 | ^[23]^ |
| Zr-doped Fe_2_O_3_ | 87.0 | 1831 | \ | 18 | ^[24]^ |
| NiCo alloy/CNTs | 75.6 | \ | \ | 16 | ^[25]^ |
| Rh/ZnO | 22.0 | 789 | 0.40 | 24 | ^[26]^ |
| CeO_2_-ZrO_2_ | 17.4 | 217.6 | 0.22 | 4 | ^[27]^ |

**Table S3.** Surface energies for various low-index crystal planes of NiOOH.

| Crystal plane | Surface energy  eV/Å^2^ |
| --- | --- |
| (001) | 0.00814 |
| (100) | 0.08676 |
| (010) | 0.09924 |

**Table S4.** Single-point energy calculations with or without dipole correction for representative and important structures and reaction steps on NiOOH.

| Structure/reaction step | Energy (eV) | | Difference  (eV) |
| --- | --- | --- | --- |
|  | without dipole correction | with dipole correction |  |
| Slab | −708.09882 | −708.09804 | 0.00078 |
| *O | −711.60261 | −711.59470 | 0.00791 |
| *OCH_3_ | −733.18398 | −733.14212 | 0.04186 |
| *OCHO + *CH_3_ | −753.59706 | −753.55987 | 0.03719 |

**Table S5.** Comparison of calculated energy barriers for the key steps of active site formation and CH_4_ activation among various catalysts.

| Catalyst | Surface | Key step | | Barrier  (eV) | Ref. |
| --- | --- | --- | --- | --- | --- |
| NiOOH | (001) | Active site formation | *OH → *O | 1.76 | This work |
|  |  | CH_4_ activation | *O → *OCH_3_ | −3.39 |  |
|  |  |  | *OCHO + * → *OCHO + *CH_3_ | −2.66 |  |
| Zr-doped Fe_2_O_3_ | (0001) | Active site formation | *OH → *O | 1.64 | ^[24]^ |
| TaHf_2_C_2_O_2_ | (0001) |  | *OH → *O | ca. 1.50 | ^[28]^ |
| Fe-N-C SAC | (001) |  | *OH → *O | 1.14 | ^[20]^ |
| Zr-doped Fe_2_O_3_ | (0001) | CH_4_ activation | *O → *CH_3_OH | −2.60 | ^[24]^ |
| TaHf_2_C_2_O_2_ | (0001) |  | CH_4_ + *O → *CH_3_OH | ca. −0.70 | ^[28]^ |
| Fe-N-C SAC | (001) |  | *O + *CH_4_ → *CH_3_OH | −1.06 | ^[20]^ |
| Fe-Ni-OH | (001) |  | *CH_4_ → *CH_3_ | −1.80 | ^[23]^ |
| NiO/Ni | (200) |  | *CH_4_ → *CH_3_ | −0.99 | ^[12]^ |
| O_v_-WO_3_ | (010) |  | *CH_4_ → *CH_3_ | 0.58 | ^[16]^ |
| O_v_-WO_3_ | (010) |  | *CH_2_OH + * → *CH_2_OH + *CH_3_ | ca. 1.20 | ^[16]^ |
| O_v_-ZnO | (100) |  | *CH_4_ → *CH_3_ | ca. 0.15 | ^[17]^ |
| Zn_v_-ZnO | (100) |  | *CH_4_ → *CH_3_ | ca. 0.30 | ^[17]^ |
| V_3_O_7_ (V site) | (200) |  | * + CH_4_ → *CH_3_ | −0.89 | ^[18]^ |
| V_3_O_7_ (O site) | (200) |  | * + CH_4_ → *CH_3_ | −1.11 | ^[18]^ |
| CuO_x_/V_2_O_5_ | \ |  | * + CH_4_ → *CH_3_ | −1.94 | ^[19]^ |
| Mo-Cu_2_Se | (111) |  | *CH_4_ → *CH_3_ | ca. −1.00 | ^[22]^ |
| Ni-Co PBA-V_CN_ | \ |  | *CH_4_ → *CH_3_ | −0.18 | ^[29]^ |

**References**

[1]	Z. Farooq, I. Shahzadi, A. Haider, H. Alhummiany, A. Ul-Hamid, W. Nabgan, M. A. Bajaber, M. Imran, M. Ikram, *Surf. Interfaces* **2023**, *43*, 103489.

[2]	S. J. Clark, M. D. Segall, C. J. Pickard, P. J. Hasnip, M. I. J. Probert, K. Refson, M. C. Payne, *Z. Kristallogr.* **2005**, *220*, 567-570.

[3]	J. P. Perdew, K. Burke, M. Ernzerhof, *Phys. Rev. Lett.* **1996**, *77*, 3865-3868.

[4]	D. Vanderbilt, *Phys. Rev. B* **1990**, *41*, 7892-7895.

[5]	R. D. King-Smith, D. Vanderbilt, *Phys. Rev. B* **1993**, *47*, 1651-1654.

[6]	R. Fletcher, *Comput. J.* **1970**, *13*, 317-322.

[7]	B. G. Pfrommer, M. Côté, S. G. Louie, M. L. Cohen, *J. Comput. Phys.* **1997**, *131*, 233-240.

[8]	S. Grimme, J. Antony, S. Ehrlich, H. Krieg, *J. Chem. Phys.* **2010**, *132*, 154104.

[9]	S. Grimme, *J. Comput. Chem.* **2006**, *27*, 1787-1799.

[10]	G. Kresse, J. Furthmüller, *Phys. Rev. B* **1996**, *54*, 11169-11186.

[11]	H. J. Monkhorst, J. D. Pack, *Phys. Rev. B* **1976**, *13*, 5188-5192.

[12]	Y. Song, Y. Zhao, G. Nan, W. Chen, Z. Guo, S. Li, Z. Tang, W. Wei, Y. Sun, *Appl. Catal. B* **2020**, *270*, 118888.

[13]	M. Chen, X. Lv, A. Guan, C. Peng, L. Qian, G. Zheng, *J. Colloid Interface Sci.* **2022**, *623*, 348-353.

[14]	Z. Guo, W. Chen, Y. Song, X. Dong, G. Li, W. Wei, Y. Sun, *Chin. J. Catal.* **2020**, *41*, 1067-1072.

[15]	A. Li, H. Qiu, Z. Wang, Y. Sun, Y. Tang, P. Wan, H. Jiang, Y. Chen, *ACS Sustain. Chem. Eng.* **2024**, *12*, 9558-9567.

[16]	J. Li, M. Luo, M. Wang, Y. Ma, G. Zheng, M. Wang, Y. Zhou, Y. Lu, C. Zhu, B. Chen, *Appl. Mater. Today* **2023**, *32*, 101855.

[17]	M. Luo, J. Li, M. Wang, Y. Ma, G. Zheng, M. Wang, Y. Zhou, *J. Environ. Chem. Eng.* **2023**, *11*, 109539.

[18]	H. Qiu, A. Li, Z. Wang, Q. Shangguan, Y. Sun, Y. Tang, P. Wan, H. Jiang, Y. Chen, *J. Colloid Interface Sci.* **2025**, *684*, 449-456.

[19]	H. Tian, Z.-Y. Zhang, H. Fang, H. Jiao, T.-T. Gao, J.-T. Yang, L. Bian, Z.-L. Wang, *Appl. Catal. B* **2024**, *351*, 124001.

[20]	C. Kim, H. Min, J. Kim, J. H. Moon, *Energy Environ. Sci.* **2023**, *16*, 3158-3165.

[21]	E. Ponticorvo, M. Iuliano, C. Cirillo, M. Sarno, *Chem. Eng. J.* **2023**, *451*, 139074.

[22]	L. Zeng, X. Wang, D. Wang, X. Peng, Z. Liu, N. Wu, K. Wang, Z. Li, B. Yang, Q. Zhang, L. Lei, P. Samorì, Y. Hou, *Energy Environ. Sci.* **2026**, *19*, 359-370.

[23]	J. Li, L. Yao, D. Wu, J. King, S. S. C. Chuang, B. Liu, Z. Peng, *Appl. Catal. B* **2022**, *316*, 121657.

[24]	J. Lee, S. Lee, C. Kim, J. S. Yoo, J. H. Moon, *Appl. Catal. B* **2024**, *344*, 123633.

[25]	J. Peng, W. Liao, X. Fu, J. Li, S. Shang, Q. Zhang, *Mol. Catal.* **2024**, *569*, 114589.

[26]	Z. Xie, M. Chen, Y. Chen, A. Guan, Q. Han, G. Zheng, *J. Phys. Chem. C* **2021**, *125*, 13324-13330.

[27]	N. B. Patricio, J. C. Cardoso, M. T. Escote, A. J. de Castro Lanfredi, A. Datye, H. Pham, C. Ribeiro, F. C. Fonseca, E. I. Santiago, *Chem. Eng. J.* **2024**, *488*, 150951.

[28]	Y. Kang, Z. Li, X. Lv, W. Song, Y. Wei, X. Zhang, J. Liu and Z. Zhao, *J. Catal.* **2021**, *393*, 20-29.

[29]	Q. Zhang, J. Peng, H. Xiong, S. Jiang, W. Li, X. Fu, S. Shang, J. Xu and G. He, *Appl. Catal. B* **2025**, *362*, 124759.
